# Evaluation of the effect of several moisturizing creams using the low frequency electrical susceptance approach

**DOI:** 10.2478/joeb-2024-0002

**Published:** 2024-02-26

**Authors:** Dindar S. Bari, Zana K. Ali, Soleen A. Hameed, Haval Y. Yacoob Aldosky

**Affiliations:** 1 Scientific Research Center, University of Zakho, Zakho, Kurdistan region, Iraq; 2 Department of Physics, College of Science, University of Zakho, Zakho, Kurdistan region, Iraq; 3 Department of Physics, College of Science, University of Duhok, Duhok, Kurdistan region, Iraq

**Keywords:** Skin, moisturizers, skin moisture, gender, age, dry

## Abstract

Moisturizers are cosmetic compounds designed to increase the moisture content of the skin. There are many types of these products in the market making it difficult for consumers to select the most effective moisturizer according to their age and gender. Hence, the aim of this study was to evaluate the effects of different moisturizers on skin hydration as well as to figure out any dependencies of the effects of these products on age or gender-related differences. We investigated the short-term moisturizing effects of five different skin moisturizers on 60 participants by using a low frequency electrical instrument. Skin surface susceptance was recorded and compared before and after the application of moisturizers. Statistically significant differences were observed in the moisturizing effect among different types of products. However, with respect to gender and age differences, there were insignificant differences in the effects of the moisturizers. Results of this study suggest that some types of moisturizers that exist in the markets are not as effective as required, which calls for a further evaluation of the moisturizers before entering markets and offering them for sale. In addition, findings suggest that gender or age differences are perhaps not important to consider in the application of moisturizers.

## Introduction

The skin forms the largest organ in the body, which weighs approximately 15% of the total body weight [1]. It has a surface area of about 1.5-2 m^2^ in adults [2], and an average thickness from 0.1 mm at its thinnest part (eyelids) to 1.5 mm or more at its thickest part (palms and soles) [1]. As the skin covers the body’s surface, it is the main interface between the body and the outside world. Therefore, it has essential vital functions such as the protection from external physical, chemical, and biological threats. In addition, the skin acts as a barrier to the passive diffusion of water out of the skin, which prevents dehydration, and has a potential role in thermoregulation [1, 3]. Moreover, the skin is also responsible for the formation of vitamin D [4].

The skin consists of multiple layers, the epidermis, the dermis, and subcutaneous tissue (hypodermis). The epidermis is the outermost layer composed of keratinized epithelial cells which function to synthesize keratin. The dermis is the skin’s middle layer between the epidermis and the subcutaneous tissue. The dermis is a tough, resilient layer and contains specialized structures, cells, ground substances, and fibers. The cells synthesize collagen and elastin fibers [3, 5]. The subcutaneous tissue is the bottom skin layer known as the fatty layer, which contains small lobes of fat cells called lipocytes [3].

The skin, like other body organs can be affected by gender-linked differences due to genetic and hormonal differences. The differences between men and women with respect to some biophysical properties of the skin are demonstrated in several studies. For example, skin pigmentation and thickness in males is greater than in females, sebum content in males is higher, in contrast, the subcutaneous fat thickness is greater in females [6-8]. However, with regard to skin elasticity, there is no significant difference between both genders [8].

The skin’s appearance and function are kept by a significant balance between the skin surface and water content of the stratum corneum (the outermost part of the epidermis). At least 10% water is required in the skin to maintain it soft and flexible. Skin is considered to be dry when its water content falls below this level. Only a 1% change in the water content of the skin can significantly alter its elasticity and permeability [9, 10]. Aging and dryness (xerosis) significantly influence the skin’s appearance and function by changing its water content. Aging causes various changes in the structural and biochemical properties as well as neurosensory perception of the skin [11]. With aging, the thickness and elasticity of the skin are reduced [12], insensible perspiration diminishes [13], the number of sweat glands and the sweat quantity is decreased [13], and skin water content is also reduced [11]. Skin dryness is a common problem and its severity increases with age. Skin dryness may impact quality of life, although it is often considered as a cosmetic problem. The most common problems of skin dryness are the possible presence of itching, reddening, or cracking [14].

There are also other factors that can play a role in dry skin such as environmental and genetic factors, and diseases. Cold or dry climates may decrease the water content of the stratum corneum, which can accentuate dry (chapped) skin. Dry skin is likely having a genetic component. The familiar tendency toward dry skin is well documented in the literature. Dry skin may also occur following diseases such as uremia or hypothyroidism. Individuals with chronic illness may also be troubled by xerosis [14].

In order to treat or fight the supposed signs of skin aging and dryness and improve skin hydration, a series of measures, known broadly as moisturizers, have been developed. Moisturizers are externally applied cosmetic compounds as a cream or lotion designed to restore moisture, increase the water content of the skin, and reduce transepidermal water loss with the aim of maintaining skin integrity and a healthy appearance. The moisturizers comprise multiple components, including humectants, which attract water from the dermis and hold it in the stratum corneum; occlusives, which provide a barrier capable of preventing transepidermal water loss in the stratum corneum; emollients, which smooth and soften skin by filling of spaces between the corneocytes; and vitamin A, E and C, which are necessary to maintain the integrity of the epithelium, the stabilization of biological membranes and collagen synthesis [14-19]. Therefore, moisturizers are a major component of skin care and are extensively used by the public daily.

A multitude of moisturizers have been designed to treat dry skin. The marketplace is flooded with many products of various components. Therefore, the choice of the most effective moisturizer might be difficult. In addition, an ideal moisturizer may be useful in one person and less useful in others to treat the common condition of skin due to variability in skin properties (physiological variations). Moreover, moisturizers are broadly promoted by different cosmetic companies with claims of strong efficacy and may enter the marketplace without proper clinical investigations [20, 21].

Therefore, this study was designed to examine the efficacy of five of the most widely used moisturizers for dry skin treatment to address the following questions: (1) Do all five moisturizers have the same efficacy once applied to the skin, (2) do all the five moisturizers have the same efficacy for both male and female, and (3) do all the five moisturizers have the same efficacy when applied on the skin of different age groups? As far as we know, there are no studies, where the efficacy of different moisturizers is evaluated, taking into account both gender and age-related differences, by assessment of skin hydration by using a low frequency, electrical method.

## Materials and methods

### Study protocol and participants

In this study, 60 healthy subjects (30 males and 30 females, mean age 39.31± 17.14 years) voluntarily participated. They were distributed over three groups based on their age as shown in Table 1. Participants were recruited from the University of Zakho. All the measurements were conducted at the University of Zakho with an ambient relative humidity in the laboratory of 30-40% and a temperature of 22-23 ^o^C. The exclusion criteria were as follows: participants with a history of skin disease or with atopic skin, receiving skin care for skin diseases, and hypersensitivity to cosmetic or skin care products were excluded from the study.

**Table 1:** Descriptive characteristics of the participants.

Group	Female	Male	Age range (years)	Mean (years)
1	10	10	18-25	21.05 ± 1.62
2	10	10	30-40	35.10 ± 2.93
3	10	10	50-70	61.80 ± 3.71

Five different skin moisturizers were acquired from local pharmacies and beauty centers, which were recommended to us and most widely used by the public. The five moisturizers were labeled as M_1_, M_2_, M_3_, M_4_, and M_5_. Five sites of the volar side of their forearm of equal (circular) areas (of diameter 2 cm) were selected and labeled 1, 2, 3, 4, and 5.

Before any measurements were undertaken, participants rested in the laboratory for at least 10 min with uncovered underarms. After that, values of skin surface susceptance were measured on the chosen skin sites before treatment with moisturizers as a control value. Then the chosen areas on the forearm of all participants were treated with moisturizers. 0.1 g of each type of moisturizer per test site was gently massaged into the circular test sites over the volar side of the forearm by using a wooden medical tongue depressor and medical gloves. Then, measurements were taken 10 min after moisturizer application. After each reading, the electrodes (that are connected to the instrument) were cleaned with sanitizing wipes and then dried with a paper towel. Thus, five different moisturizers were tested along with one non-treated reference area.

At the end of all recordings, redundant materials were removed from the underarms of the participants with sanitizing wipes and a paper towel. During the whole period of measurements, all participants sat comfortably in a chair and they were not allowed to do any other activity.

### Instrumentation

In the current work, the Sensoderm mod. 960 from Skinstrument AS, Norway was used for non-invasive measurements of the skin surface susceptance in units of μS/cm^2^, which is directly related to the skin moisture [22, 23]. The device works by applying a small AC voltage of about 60 mV rms (not percepted by the participants) with a three- electrode system to the skin and measures the electrical susceptance at 88 Hz [23]. This approach is recommended and it is found to be suitable for skin moisture assessment in several studies (see e.g. [23-28]). The employed device uses low frequency within a range of 20 Hz to a few hundred hertz, which is the required value for ensuring that the measurements are only focused on the stratum corneum [29, 30]. In this way, the contribution from viable skin layers such as the dermis was reduced. Moreover, focusing on measuring only the electrical susceptance and neglecting conductance is done to eliminate the contribution from sweat duct filling since the conductance value is strongly influenced by the sweat level in the ducts. Electrical susceptance, on the other hand, is strongly dependent on the moisture content of the stratum corneum and it is not influenced by sweat duct activity [31]. Hence, any variation that occurs in skin moisture will lead to changes in susceptance. Skin susceptance (moisture) recordings were done by pressing a spring-loaded probe against the skin and then the measured value was displayed on the instrument after 5 seconds.

### Statistical analysis

To analyze the differences among five types (groups) of moisturizers and compare them to a control site, and analyze the effects of age, and compare the three age groups, oneway repeated analysis of variation (ANOVA) was utilized followed by multiple pairwise comparisons tests using Sidak correction. Finally, to assess the effect of gender (male vs. female), the Mann-Whitney U test was used. All the statistical analyses were performed by using IBM SPSS Statistics.

### Informed consent

Informed consent has been obtained from all individuals included in this study.

### Ethical approval

The protocol has been complied with all relevant national regulations, institutional policies and in accordance with the tenets of the Helsinki Declaration, and has been approved by the authors’ institutional review board or equivalent committee.

## Results

### Skin surface susceptance as a function of different moisturizers

**Fig. 1:**
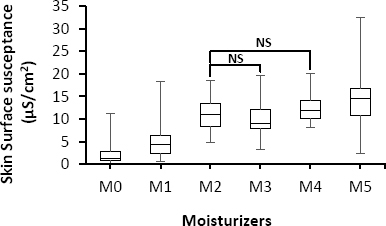
Box-plot with medians, quartiles, and the min and max as whiskers, showing variation in skin surface susceptance in relation to the five different types of moisturizers, NS = *p* > 0.05.

Figure 1 shows the median values of skin surface susceptance with respect to the base and with different types of moisturizers. Based on these results and ANOVA analysis, all five moisturizers highly significantly (*p*<0.0001) increased skin surface susceptance. In addition, results of post hoc multiple comparisons showed significant differences (*p*<0.0001) among various types of moisturizers except between type two (M_2_) on one hand and type three (M_3_) and type four (M_4_) on the other hand. The error bars seen in the figure indicate the maximum and minimum skin surface susceptance values obtained from the participants (n=60). The large error levels shown point to individual differences. Table 2 presents the percentage of increases in skin susceptance, which is related to an increase in hydration after treatment of the skin with the five moisturizers. Based on these findings, the moisturizer type five (M_5_) led to the highest (1349.17%) increase in skin surface susceptance (i.e., moisture), whereas the moisturizer type one (M_1_) produced the lowest (289.72%) increase in skin surface susceptance compared to other moisturizer types.

**Table 2:** Percentage of increases in skin surface susceptance after application of five different moisturizers.

M_1_/M_0_	M_2_/M_0_	M_3_/M_0_	M_4_/M_0_	M_5_/M_0_
289.72	1074.82	935.08	1245.10	1349.17

### Skin surface susceptance as a function of age for different moisturizers

**Fig. 2:**
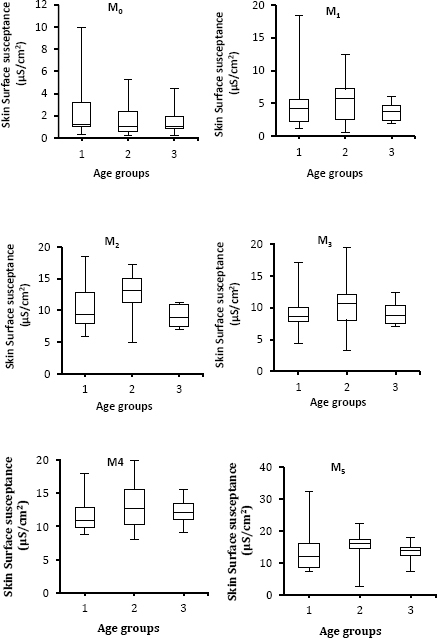
Boxplots with medians, quartiles, and the min and max as whiskers, showing variation in skin surface susceptance in relation to the different age groups for different moisturizers.

Figure 2 shows changes in median values of skin surface susceptance with respect to the different age groups and for the five different moisturizers. One can see that the median values of skin surface susceptance of the three groups are increased following the application of different moisturizers. In addition, group two is more influenced by moisturizers in contrast to the other two groups. However, a nonsignificant (*p*> 0.05) difference between the age groups was observed as indicated by the ANOVA test. The error bars in the figure represent the variability of data and are used to indicate the variations within each group (n=20) in skin surface susceptance measurement.

### Skin surface susceptance as a function of gender for different moisturizers

**Fig. 3:**
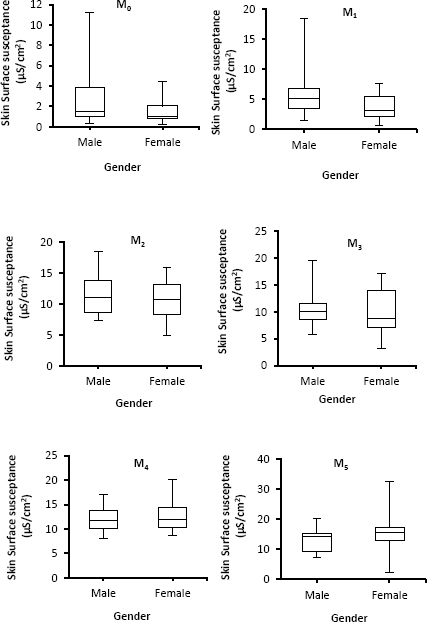
Boxplots with medians, quartiles, and the min and max as whiskers, showing variation in skin surface susceptance in relation to the different genders for different moisturizers.

Data presented in Figure 3, show that the skin of both males and females was hydrated after applications of moisturizers. In addition, males’ skin surface susceptance is higher than that of females. However, when moisturizers type four (M_4_) and type five (M_5_) were applied to the skin of both genders, female skin surface susceptance was slightly higher than that of males, which is opposite to the action of the other three types of moisturizers where male skin surface susceptance was higher. When these findings were statistically analyzed using the Mann-Whitney U test insignificant differences (*p* > 0.05) were observed between both groups (males and females). The error bars in Figure 3 are the maximum and minimum of skin surface susceptance among both genders and point to differences between subjects (n=30) within each group.

## Discussion

This study was conducted to investigate the efficacy of different moisturizing creams on skin surface susceptance, which is correlated with the skin moisture of different age groups and genders by using the low-frequency electrical susceptance method. In this study, we assessed the efficacy of moisturizers by their short-term efficacy as performed in some published works [32, 33]. Generally, the study results showed that there are significant differences in the effect of various skin moisturizers. In addition, there were insignificant differences in the efficacy of moisturizers with respect to age and gender differences after a single application of moisturizer.

A small (0.1 g) single application of moisturizers induced variations in the electrical properties of the stratum corneum, increasing electrical capacitance as assessed by skin susceptance. In addition, in this study we used a low frequency (< 1 kHz) susceptance method, which is proven to be an appropriate method for assessing skin hydration [24, 25, 29, 34]. All the moisturizers used significantly improved skin surface susceptance compared to baseline measurements (Figure 1 and Table 2). However, the efficacy of the moisturizers was different according to the skin surface susceptance measurements obtained from the test subjects, which is in line with the findings of [21, 23]. This could be due to the fact that some available moisturizers on the market are inappropriately working or could also be due to differences in skin types and suggests that one particular moisturizer may be ideal for one skin type but inefficient for another type [23].

The study findings reveal that age-related differences have a small insignificant impact on the efficacy of the moisturizers. Moreover, the skin of the middle-aged group (30-40 years) appear more hydrated after applications of moisturizers compared to the other two groups, but not statistically significant. Interpretation of these results may reveal that the used moisturizers had the same effectiveness for the three age groups. In other words, the moisturizers improved the moisture content of the skin over the baseline for the age groups without showing significant differences between age-groups. Another possibility of explaining the lack of statistical significance of age-related variations in skin surface susceptance (moisture) could be due to the small sample size (n=20 for each group), and low power in detecting small differences at the group level. Furthermore, small and insignificant differences among groups with respect to skin surface susceptance in this study might also be due to the fact that the measurements were done 10 min (i.e., short-term) after applications of moisturizers. These results are in line with [35], who could not demonstrate statistically significant differences in moisturizer effects with respect to age-related differences among infants and toddlers.

Moisturizers increase the skin surface susceptance of both genders because of improved skin hydration. As with age, there were insignificant differences between males and females with respect to the efficacy of different moisturizers, although small differences were observed. These results are in agreement with [35], who also found the same results with infants and toddler participants. These findings reflected a consistent effect of moisturizer on the skin of both genders irrespective of differences by gender. In other words, this indicates that the employed moisturizers increased the moisture content or hydration of the corneum for both genders without taking physiological differences into account. In addition, even though the skin of males in general in all anatomic locations is thicker than females [36], this did not lead to significant differences in skin moisture (as assessed by skin surface susceptance) of both genders after the application of five different moisturizers.

## Conclusion

Our findings clearly illustrate that there are significant differences between the effects of different skin moisturizers, in which some of them increased the skin hydration of participants much more than others. This indicates that some moisturizers available in markets are more effective than others. This requires relevant authorities to further evaluate moisturizers before recommending their use and also to evaluate customers’ skin to reveal the best types of products for them. According to this study, age and gender differences did not affect the efficiency of any cream used to hydrate the skin. It implies that grouping moisturizers according to demographic distinctions is not crucial.
